# Anti-Inflammatory Effects of Paraprobiotic *Lactiplantibacillus plantarum* KU15122 in LPS-Induced RAW 264.7 Cells

**DOI:** 10.4014/jmb.2404.04052

**Published:** 2024-06-03

**Authors:** Hye-Won Lee, Hee-Su Jung, Na-Kyoung Lee, Hyun-Dong Paik

**Affiliations:** Department of Food Science and Biotechnology of Animal Resources, Konkuk University, Seoul 05029, Republic of Korea

**Keywords:** *Lactiplantibacillus plantarum*, anti-inflammatory, paraprobiotics, NF-κB signaling pathway, MAPK signaling pathway

## Abstract

Inflammation is a biodefense mechanism that provides protection against painful conditions such as inflammatory bowel disease, other gastrointestinal problems, and irritable bowel syndrome. Paraprobiotics have probiotic characteristics of intestinal modulation along with merits of safety and stability. In this study, heat-killed *Lactiplantibacillus plantarum* KU15122 (KU15122) was investigated for its anti-inflammatory properties. KU15122 was subjected to heat-killed treatment for enhancement of its safety, and its concentration was set at 8 log CFU/mL for conducting different experiments. Nitric oxide production was most remarkably reduced in the KU15122 group, whereas it was increased in the LPS-treated group. In RAW 264.7 cells, KU15122 inhibited the expression of inducible nitric oxide synthase, cyclooxygenase-2, interleukin (IL)-1β, IL-6, and tumor necrosis factor-α. ELISA revealed that among the tested strains, KU15122 exhibited the most significant reduction in PGE_2_, IL-1β, and IL-6. Moreover, KU15122 inhibited various factors involved in the nuclear factor-kappa B, activator protein-1, and mitogen-activated protein kinase pathways. In addition, KU15122 reduced the generation of reactive oxygen species. The anti-inflammatory effect of KU15122 was likely attributable to the bacterial exopolysaccharides. Conclusively, KU15122 exhibits anti-inflammatory potential against inflammatory diseases.

## Introduction

*Lactiplantibacillus plantarum* stands as a significant species among the lactic acid bacteria (LAB) and possesses varied probiotic characteristics [1]. *L. plantarum* is present in dairy products, fermented foods, and the mouth and intestinal tract of the host. *L. plantarum* is effective in treating various health conditions, including controlling the composition of fecal flora and preventing and treating irritable bowel syndrome (IBS), inflammatory bowel disease (IBD), coronary heart disease, cancer, and other gastrointestinal problems [2]. Non-viable microbial cells, often referred to as paraprobiotics, are safer and more stable than live probiotics. Heat-killed LAB can diminish the hazard of microbial infection and translocation of antibiotics resistance and are easier to store [3].

Excessive lipopolysaccharides (LPS) can activate inflammation-related cellular signaling pathways, including nuclear factor-kappa B (NF-κB), activator protein-1 (AP-1), and mitogen-activated protein kinases (MAPKs) [4]. NF-κB family members regulate the functioning of several proinflammatory cytokines, transcription factors, cell surface receptors, and adhesion molecules, which play major roles in intestinal inflammation [5]. Activated inflammatory cells produce additional cytokines such as tumor necrosis factor-α (TNF-α), interleukin-(IL)-1β, and IL-6 along with nitric oxide (NO), prostaglandin E2 (PGE_2_) [6]. NO and PGE_2_ are essential proinflammatory agents produced by iNOS and COX-2, respectively [7]. In response to proinflammatory cytokines, MAPKs facilitate the transcription and activation of diverse transcription factors that control genes associated with IBD, and elevated levels of MAPK expression have been observed in individuals with IBD [8]. Primary activation of AP-1 occurs via MAPKs, including extracellular signal-regulated kinase (ERK1/2), c-Jun N-terminal kinase (JNK), and p38 [9].

Reactive oxygen species (ROS) play a key role in multiple physiological functions and are triggered by LPS. Oxidative stress arises due to an disparity between the generation of free radicals and development of various biological conditions such as arthritis, chronic abdominal pain, cancer, and IBD [2]. Individuals with IBS show decreased antioxidant capacity as a result of increased ROS, and alterations in the enzymatic system responsible for oxidative stress management may be involved in the development of IBS and its symptoms [10].

The objective of this study was to demonstrate the anti-inflammatory effect of *L. plantarum* KU15122 by inducing anti-inflammatory cytokines and suppressing proinflammatory cytokines and ROS in RAW 264.7 cells. In addition, involvement of the NF-κB, AP-1, and MAPK signaling pathways was confirmed.

## Material and Methods

### Sample Preparation

*L. plantarum* KU15122 was isolated from kimchi, a traditional Korean fermented food. *L. plantarum* ATCC 14917 and *Lacticaseibacillus rhamnosus* GG (LGG) were acquired from the Korean Collection for Type Cultures (KCTC; Republic of Korea) and used as comparative strains. LAB strains were cultured in De Man–Rogosa–Sharpe (MRS) (BD Difco, USA) broth at 37°C for 24 h. To collect the cells, bacterial cultures underwent at 14,240 ×*g* at 4°C for 5 min. LAB were cleaned twice with phosphate-buffered saline (PBS) and resuspended in Dulbecco’s modified Eagle’s medium (DMEM) (Hyclone, USA). Whole LAB heated at 80°C for 30 min in water bath (Hanil Scientific Inc., Republic of Korea) to render them non-viable. Heat-killed bacterial cells were utilized at a concentration of 8 log CFU/ml.

### Cell Culture

The murine macrophage RAW 264.7 cell line (KCLB 40071) was obtained from the Korean Cell Line Bank (KCLB; Republic of Korea). RAW 264.7 cells were seeded in DMEM supplemented with 1% streptomycin/penicillin solution and 10% fetal bovine serum (FBS; Hyclone). The cells were incubated in a 5% CO_2_ incubator at 37°C (Sanyo, Japan).

### Cell Viability

Thiazolyl blue tetrazolium bromide (MTT) assay was used to evaluated the viability of RAW 264.7 cells [11]. RAW 264.7 cells (2 × 10^5^ cells/well) were placed into 96-well plates and incubated for 2 h, followed by the addition of heat-killed LAB. After 24 h, the supernatant was eliminated, and the cells were cleaned twice with PBS. Subsequently, MTT solution (0.5 mg/ml) was treated to each well, and the cells were incubated for 1 h. The liquid above was taken out, and the formazan crystals were dispersed using dimethyl sulfoxide. Using a microplate reader, the absorbance at 570 nm was determined.

### LPS-induced NO Production

Cells were plated at a concentration of 2 × 10^5^ cells/well in 96-well culture plates and cultured for 2 h [12]. LPS (1 μg/ml, Sigma-Aldrich, USA) was used as the positive control of the experiment. After treatment, all samples were incubated for 24 h, and 100 μl of supernatant without cells were mixed with 100 μl of Griess solution in plates for a duration of 15 min. The absorbance was assessed at 540 nm for estimation of NO production using sodium nitrite standard curve.

### Quantification of Cytokine Gene Expression

Real-Time Polymerase Chain Reaction (qRT-PCR) was employed based on a prior study's methodology, incorporating certain adaptations [13]. RAW 264.7 cells were seeded in a 6-well plate (1 × 10^6^ cells/well) cultured for 24 h, and treated with heat-killed LAB (1 × 10^8^ CFU/well). After 2 h, LPS treatment (1 μg/ml) was performed. The total RNA was extracted using the RNeasy Mini Kit (Qiagen, Germany). cDNA was generated using the RevertAid First Strand cDNA Synthesis Kit (Bioline, UK). qRT-PCR was conducted by blending cDNA with SYBR Green PCR Master mix and primers. The primers used were as follows: β-actin: forward 5'-GTGGGCCGCCCTAGGCACCAG-3' and reverse 5'-GGAGGAAGAGGATGCGGCAGT-3’; iNOS: forward 5'-CCCTTCCGAAGTTTCTGGCAGCAGC-3' and reverse 5'-GGCTGTCAGAGCCTCG-TGGCTTTGG-3'; COX-2: forward 5'-CACTACATCCTGACCCACTT-3' and reverse 5'-ATGCTCCTGCTTGAGTATGT-3'; IL-1β: forward 5'-CAGGATGAGGACATGAGCACC-3' and reverse 5'-CTCTGCAGACTCAAACTCCAC-3'; IL-6: forward 5'-GTACTCCAGAAGACCAGAGG-3' and reverse 5'-TGCTGGTGACAACCACGGCC-3'; TNF-α: forward 5'-TTGACCTCAGCGCTGAGTTG-3' and reverse 5'-CCTGTAGCCCACGTCGTAGC-3’ [14]. RT-PCR assay conditions were programmed as follows: 95°C for 2 min for polymerase activation, followed by 40 cycles of 95°C for 20 s, 65°C for 20 s, and 72°C for 30 s. The cycle threshold (Ct) value was normalized to that of the housekeeping gene β-actin. The relative gene expression level was evaluated using the 2^-ΔΔCt^ method.

### Cytokine and PGE_2_ Production Using ELISA

RAW 264.7 cells were placed a concentration of 5 × 10^5^ cells/well in 12-well plates. After 2 h, heat-killed LAB were treated with LPS (1 μg/ml) for 24 h, and the concentrations of PGE_2_, IL-1β, and IL-6 in the culture medium were estimated following the manufacturer’s instructions. Using an ELISA kit (Thermo Fisher Scientific, USA; R&D Systems, USA), the levels of PGE_2_ , IL-1β, and IL-6 were assessed.

### Signaling Pathway Analysis Using Western Blotting

RAW 264.7 cells were seeded in a 6-well plate (4 × 10^6^ cells/well) overnight, and the samples were treated with LPS (1 μg/ml). Total protein was isolated from RAW 264.7 cells using lysis buffer (iNtRON Biotechnology, Republic of Korea) with a protease/phosphatase inhibitors. Twenty micrograms of each protein were fractionated using 10% sodium dodecyl sulfate-polyacrylamide gel electrophoresis (SDS-PAGE) and moved onto a polyvinylidene fluoride (PVDF) membrane [15]. Membranes were blocked with 5% skim milk in Tris-buffered saline with 1% Tween 20 (TBST) for 1 h, and were incubated with specific primary antibodies GAPDH (control), p38, p-p38, JNK, p-JNK, c-Jun, p-c-Jun, ERK, p-ERK, p65, p-p65, and IκB-α (Cell Signaling Technology Inc., USA) at 4°C for 16–24 h. After washing with TBST, the membranes were displayed to horseradish peroxidase-conjugated secondary antibodies (Cell Signaling Technology Inc.) for 1 h. Following a rinse with TBST, protein bands were identified using an improved chemiluminescence solution, and images were taken by displaying PVDF membranes to X-ray film.

### ROS Production through Staining

RAW 264.7 cells (5 × 10^5^ cells/well) were seeded into 12-well plates and cultured at 37°C [16]. After 2 h, the cells were added samples and cultured with 1 μg/ml LPS for 18–24 h. Before removing the media, the wells were scrubbed twice with PBS. Each well was exposed with 20 μM 2',7'-Dichlorodihydrofluorescein diacetate (DCFH-DA) (Sigma-Aldrich) and left undisturbed for 40 min in the darkroom. Images were captured using a DS-Ri2 digital camera (Nikon Co. Ltd., Japan) after the cells were observed under a fluorescence microscope (Nikon Co. Ltd.).

### Production and Separation of Exopolysaccharides

The EPS obtained from each sample was purified using the ethanol precipitation [17]. The bacterial suspension was centrifuged at 10,000 ×*g* for 10 min to acquire cell precipitates, which were then rinsed twice with 0.9% NaCl and centrifuged again. In the physical extraction method, cleaned bacterial pellets were reconstituted in 1 M NaCl, sonicated in a QSonica sonicator (USA) at 40 W for 3 min, and maintained on ice. Subsequently, supernatants of each treatment were acquired by centrifugation at 10,000 ×*g* for 10 min, then blended with twice their volume of ethanol and left to rest overnight at 4°C. The resulting EPS were collected after centrifugation at 10,000 ×*g* for 20 min and dissolved in ddH_2_O. The solution was stored at −80°C.

The dissolved EPS extract was evaluated using the phenol-sulfate method. A combination of EPS solution, 5%phenol, and sulfuric acid was prepared, and the presence of polysaccharides in the extract was indicated by an observable color reaction [18].

### Statistical Analysis

Every experiments were examined in triplicate, and results are represented as the mean ± standard deviation. A difference of means was conducted using one-way analysis of variance (ANOVA), where significance was determined at *p* < 0.05. Statistical analyses were performed using SPSS software (version 18.0; SPSS Inc., USA).

## Results

### Effects of Heat-Killed *L. plantarum* KU15122 on Cell Viability and NO Production

**Fig. 1:**
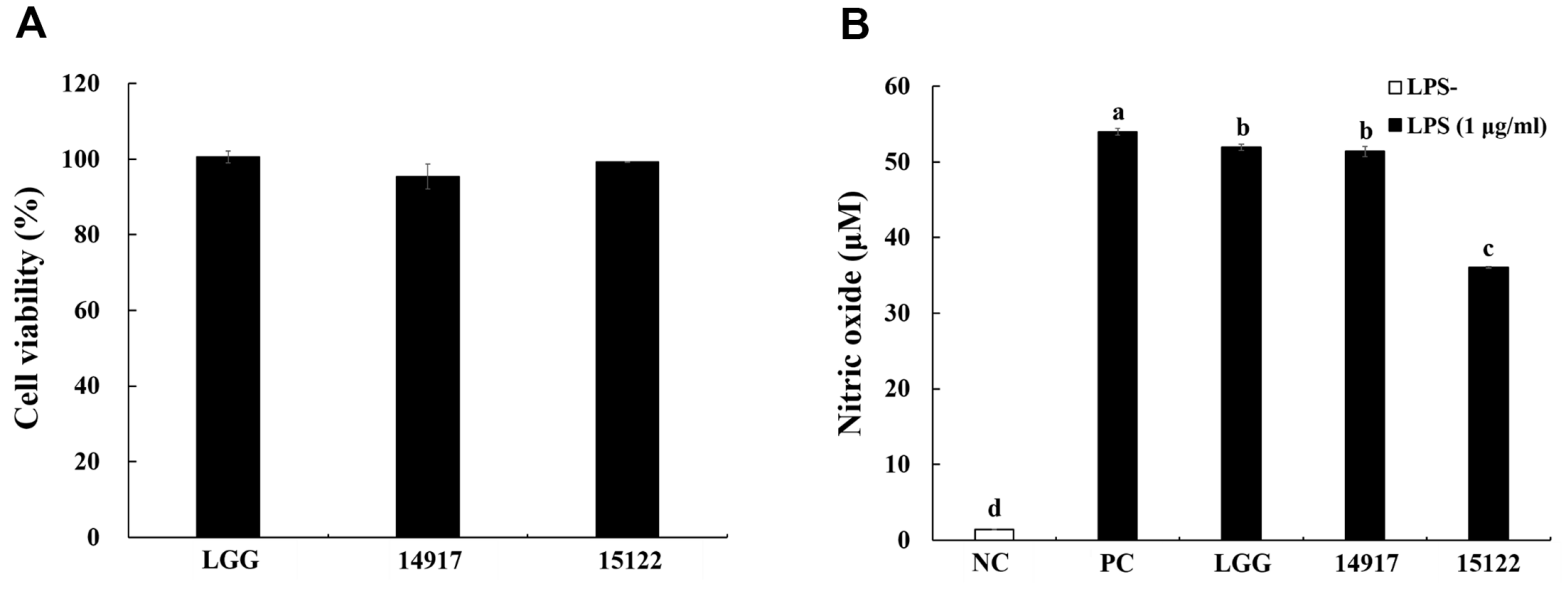
Effects of heat-killed LAB strains on cell viability and nitric oxide (**NO**) production in LPS-induced RAW 264.7 cells. (**A**) Cell viability, (**B**) NO production. NC, negative control without LPS; PC, positive control with LPS; LGG, *L. rhamnosus* GG; 14917, *L. plantarum* ATCC 14917; 15122, *L. plantarum* KU15122. Data are presented as mean ± standard deviation of triplicate experiments. Different letters on error bars represent significant differences (*p* < 0.05).

RAW 264.7 cells were used to access the effect of heat-killed *L. plantarum* KU15122 on cell viability to exclude that its anti-inflammatory properties are related to cytotoxicity. Heat-killed LGG, *L. plantarum* ATCC 14917, and *L. plantarum* KU15122 had no noticeable effects on cell viability at any of the concentrations tested (Fig. 1A). Proceeding with subsequent experiments using concentrations of heat-killed *L. plantarum* KU15122 that exhibited no cytotoxicity, ensuring no impact on its anti-inflammatory properties.

To assess the anti-inflammatory capacity of heat-killed *L. plantarum* KU15122, the effect of *L. plantarum* KU15122 on NO production in LPS-induced RAW 264.7 cells was examined. In the LPS-treated group, NO production was significantly higher compared to the LPS-negative group. In both the 8 log CFU/ml and 9 log CFU/ml, NO production were observed without cytotoxicity (data not shown). However, this study dealt on the overall mechanism at just 8 log CFU/ml. At 8 log CFU/ml, treatment with heat-killed *L. plantarum* KU15122 significantly inhibited NO production. Additionally, *L. plantarum* KU15122 exhibited lower NO production than that of LGG and *L. plantarum* ATCC 14917 (Fig. 1B).

### Effect of Heat-Killed *L. plantarum* KU15122 on mRNA Expression of iNOS, COX-2, and Proinflammatory Cytokines

**Fig. 2:**
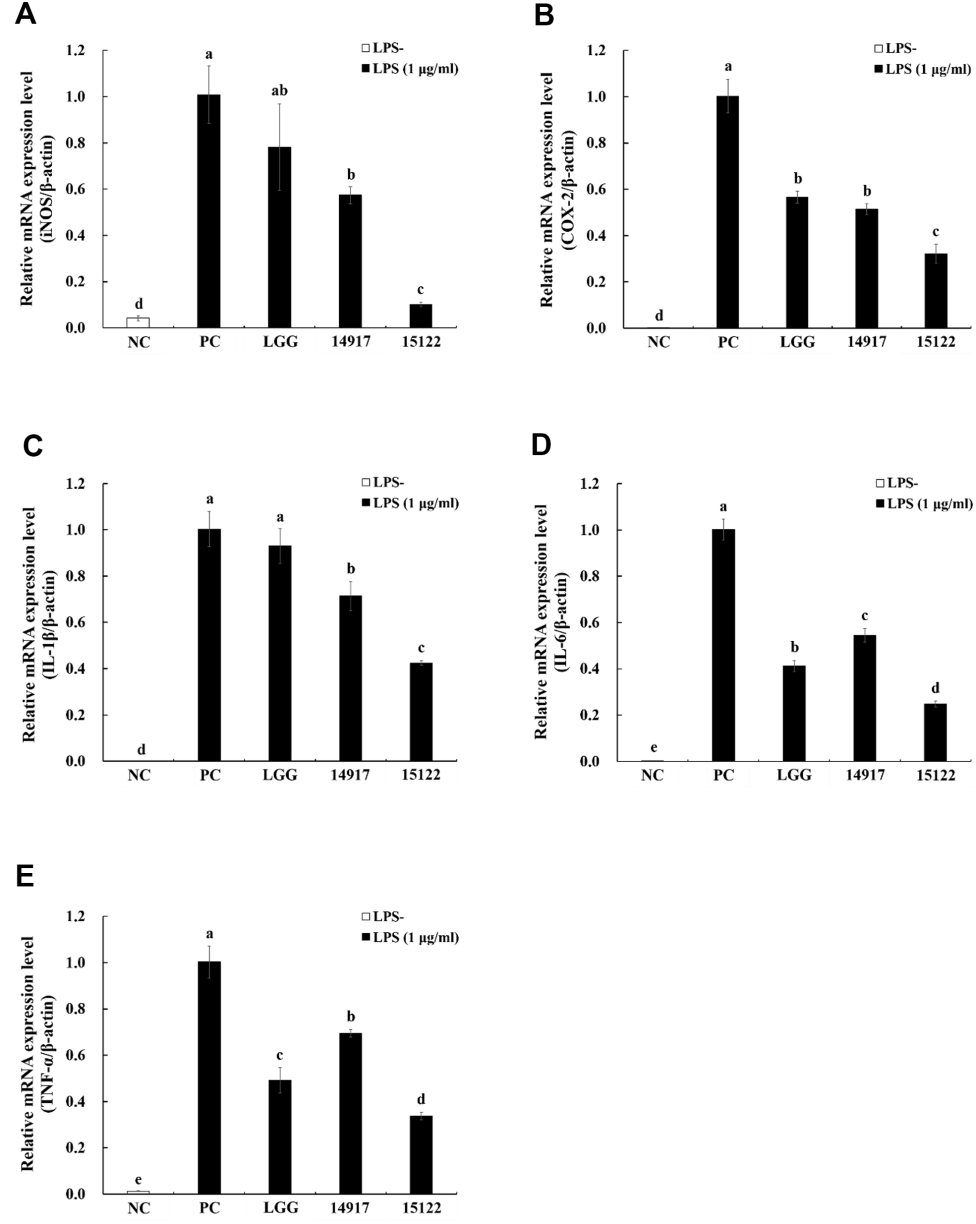
Effects of heat-killed LAB strains on mRNA expression of proinflammatory factors and proinflammatory cytokines in LPS-induced RAW 264.7 cells. (**A**) iNOS, (**B**) COX-2, (**C**) IL-1β, (**D**) IL-6, (**E**) TNF-α. NC, negative control without LPS; PC, positive control with LPS; LGG, *L. rhamnosus* GG; 14917, *L. plantarum* ATCC 14917; 15122, *L. plantarum* KU15122. Data are presented as mean ± standard deviation of triplicate experiments. Different letters on error bars represent significant differences (*p* < 0.05).

RT-PCR was performed to explore whether heat-killed *L. plantarum* KU15122 decreased the mRNA expression of iNOS, COX-2, and proinflammatory cytokines. In contrast to the negative group, LPS treatment resulted in notably elevated levels of iNOS, COX-2, IL-1β, IL-6, and TNF-α (Fig. 2A-2E). However, heat-killed *L. plantarum* KU15122 group showed the lowest levels of iNOS, COX-2, and proinflammatory cytokines.

### Effect of Heat-Killed *L. plantarum* KU15122 on Protein Levels of PGE_2_, IL-1β, and IL-6

**Fig. 3:**
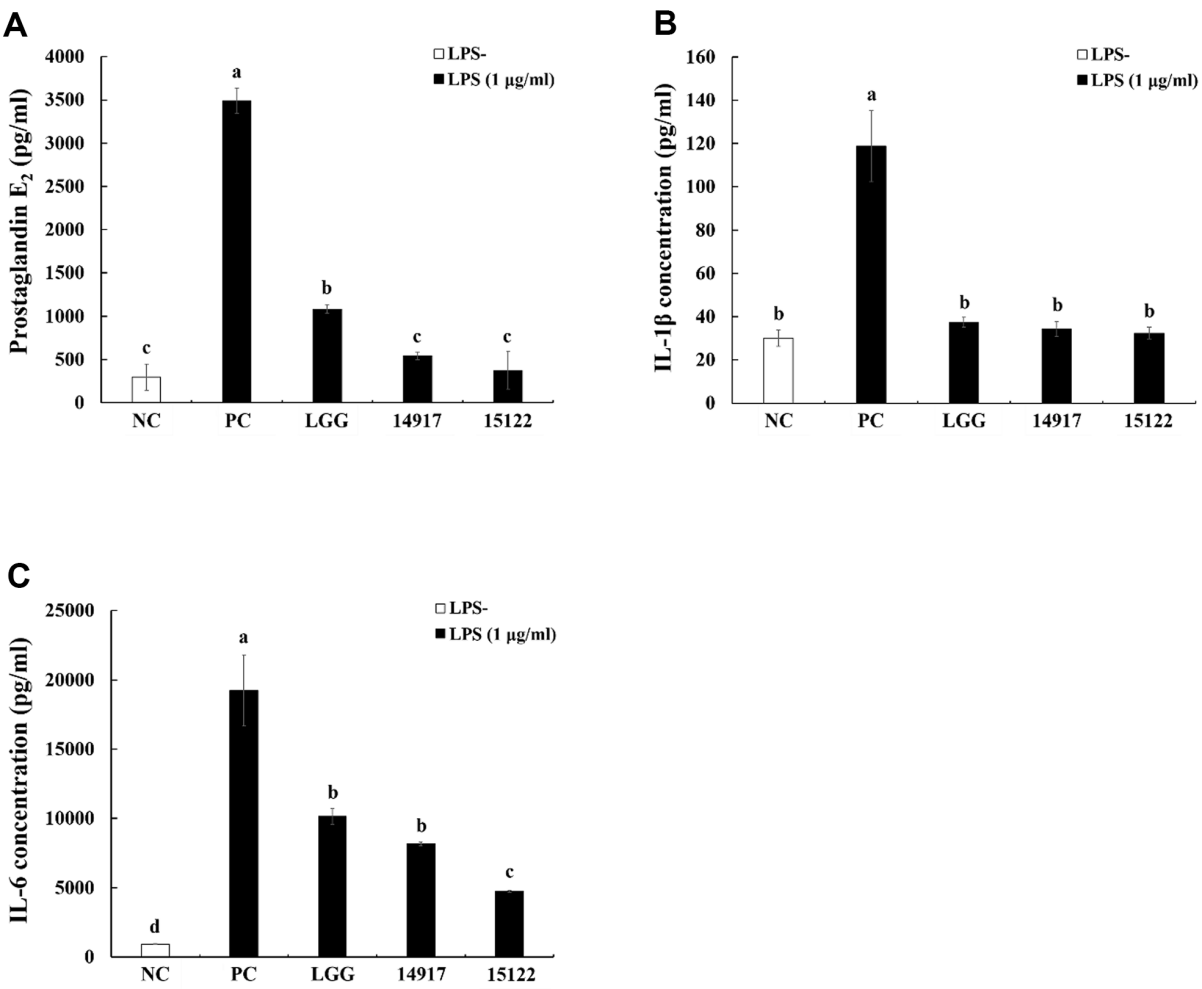
Effects of heat-killed LAB strains on protein levels of PGE_2_, IL-1β, and IL-6 in LPS-induced RAW 264.7 cells. (**A**) PGE_2_, (**B**) IL-1β, (**C**) IL-6. NC, negative control without LPS; PC, positive control with LPS; LGG, *L. rhamnosus* GG; 14917, *L. plantarum* ATCC 14917; 15122, *L. plantarum* KU15122. Data are presented as mean ± standard deviation of triplicate experiments. Different letters on error bars represent significant differences (*p* < 0.05).

As per ELISA results, LPS activation notably prompted a significant rise in the transcriptional presence of PGE_2_, IL-1β, and IL-6. In contrast, heat-killed *L. plantarum* KU15122 treatment led to a reduction in the protein levels of PGE_2_, IL-1β, and IL-6 (Fig. 3A-3C). Compared with LGG and *L. plantarum* ATCC 14917, *L. plantarum* KU15122 showed similar or greater reduction in protein expression.

### Effect of Heat-Killed *L. plantarum* KU15122 on NF-κB and AP-1 Signaling

**Fig. 4:**
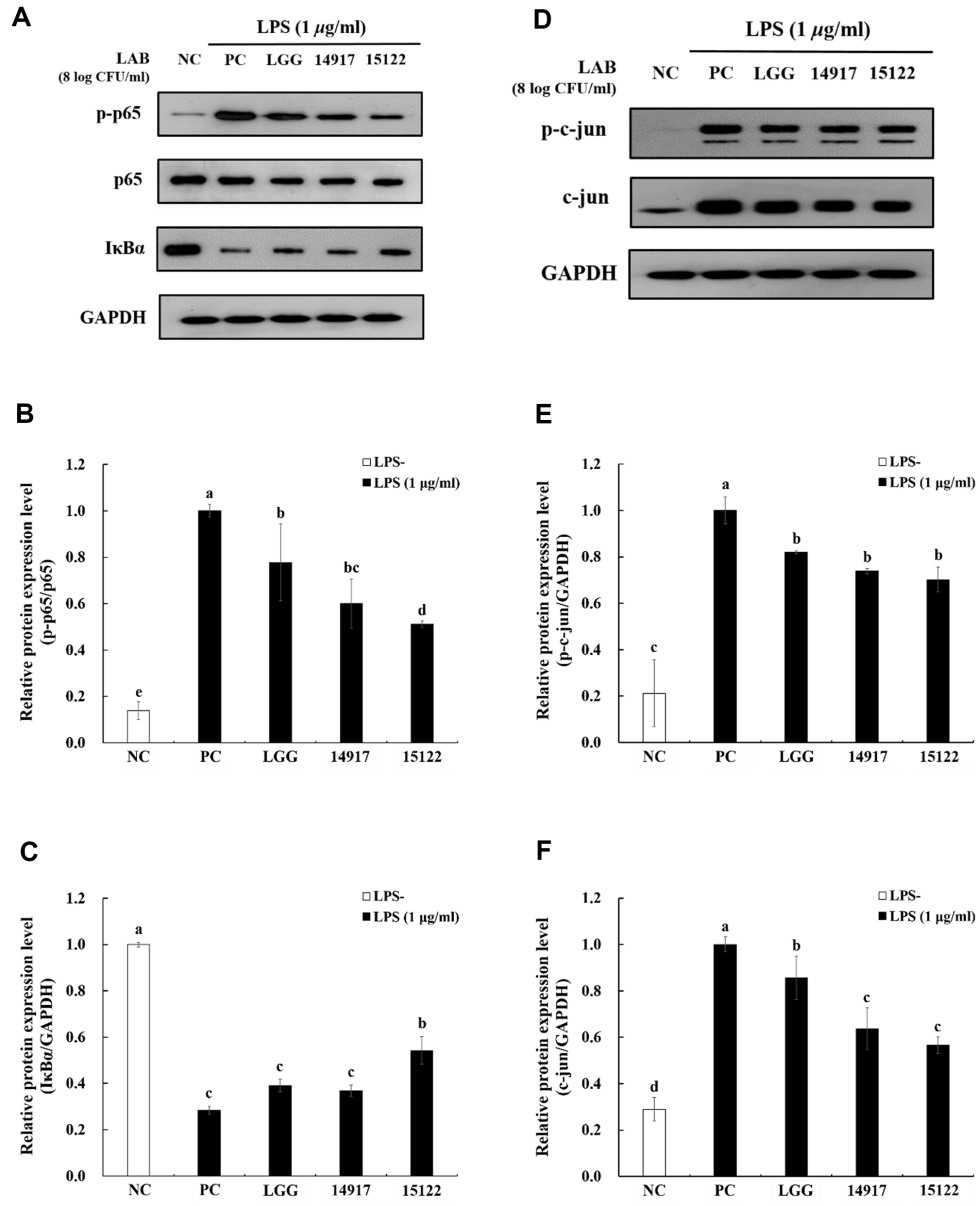
Effects of heat-killed LAB strains on NF-κB and AP-1 activation in LPS-induced RAW 264.7 cells. (**A**) analysis of NF-κB pathway, (**B**) p-p65/p65, (**C**) IκBα/GAPDH, (**D**) analysis of AP-1 pathway, (**E**) p-c-Jun/GAPDH, (**F**) c-Jun/ GAPDH. NC, negative control without LPS; PC, positive control with LPS; LGG, *L. rhamnosus* GG; 14917, *L. plantarum* ATCC 14917; 15122, *L. plantarum* KU15122. Data are presented as mean ± standard deviation of triplicate experiments. Different letters on error bars represent significant differences (*p* < 0.05).

To determine whether downregulation of proinflammatory factors was accompanied, the effect of heat-killed *L. plantarum* KU15122 on NF-κB and AP-1 signaling was assessed in LPS-induced RAW 264.7 cells. LPS stimulation led to marked phosphorylation of NF-κB such as IκB-α and p-p65. Compared with the positive control treated with LPS, reduction in phosphorylation of IκB-α and p65 expression was observed in the *L. plantarum* KU15122 group, as shown in Fig. 4A-4C. As Fig. 4D-4F, the expression of p-c-Jun was reduced by *L. plantarum* KU15122 compared to the control group treated with LPS. These results indicate that *L. plantarum* KU15122 inhibited the inflammatory reaction by modulating the AP-1 and NF-κB signaling pathways.

### Effect of Heat-Killed *L. plantarum* KU15122 on MAPK Signaling

**Fig. 5:**
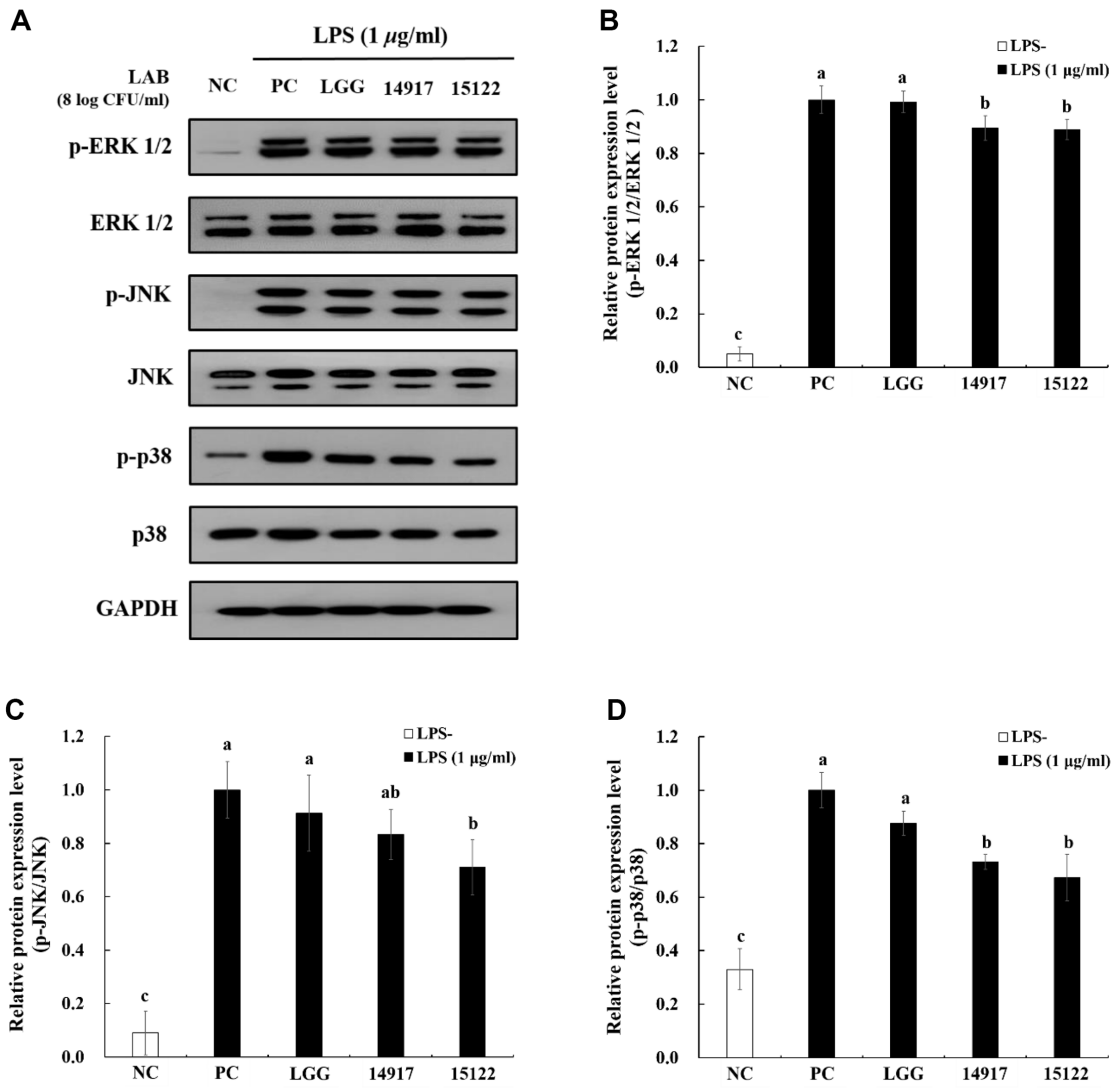
Effect of heat-killed LAB strains on the MAPK pathway activation in LPS-induced RAW 264.7 cells. (**A**) analysis of MAPK pathway, (**B**) p-ERK1/2/ERK1/2, (**C**) p-JNK/JNK, (**D**) p-p38/p38. NC, negative control without LPS; PC, positive control with LPS; LGG, *L. rhamnosus* GG; 14917, *L. plantarum* ATCC 14917; 15122, *L. plantarum* KU15122. Data are presented as mean ± standard deviation of triplicate experiments. Different letters on error bars represent significant differences (*p* < 0.05).

In response to LPS stimulation, MAPKs such as ERK 1/2, JNK, and p38 were markedly phosphorylated (Fig. 5A-5D). In contrast, heat-killed *L. plantarum* KU15122 exhibited a decrease in p-ERK 1/2, p-p38, and p-JNK, demonstrating its anti-inflammatory effects. These results revealed that *L. plantarum* KU15122 mediated anti-inflammatory properties by inhibiting MAPK activation.

### Effect of Heat-Killed *L. plantarum* on ROS Production in RAW 264.7 Cells

**Fig. 6:**
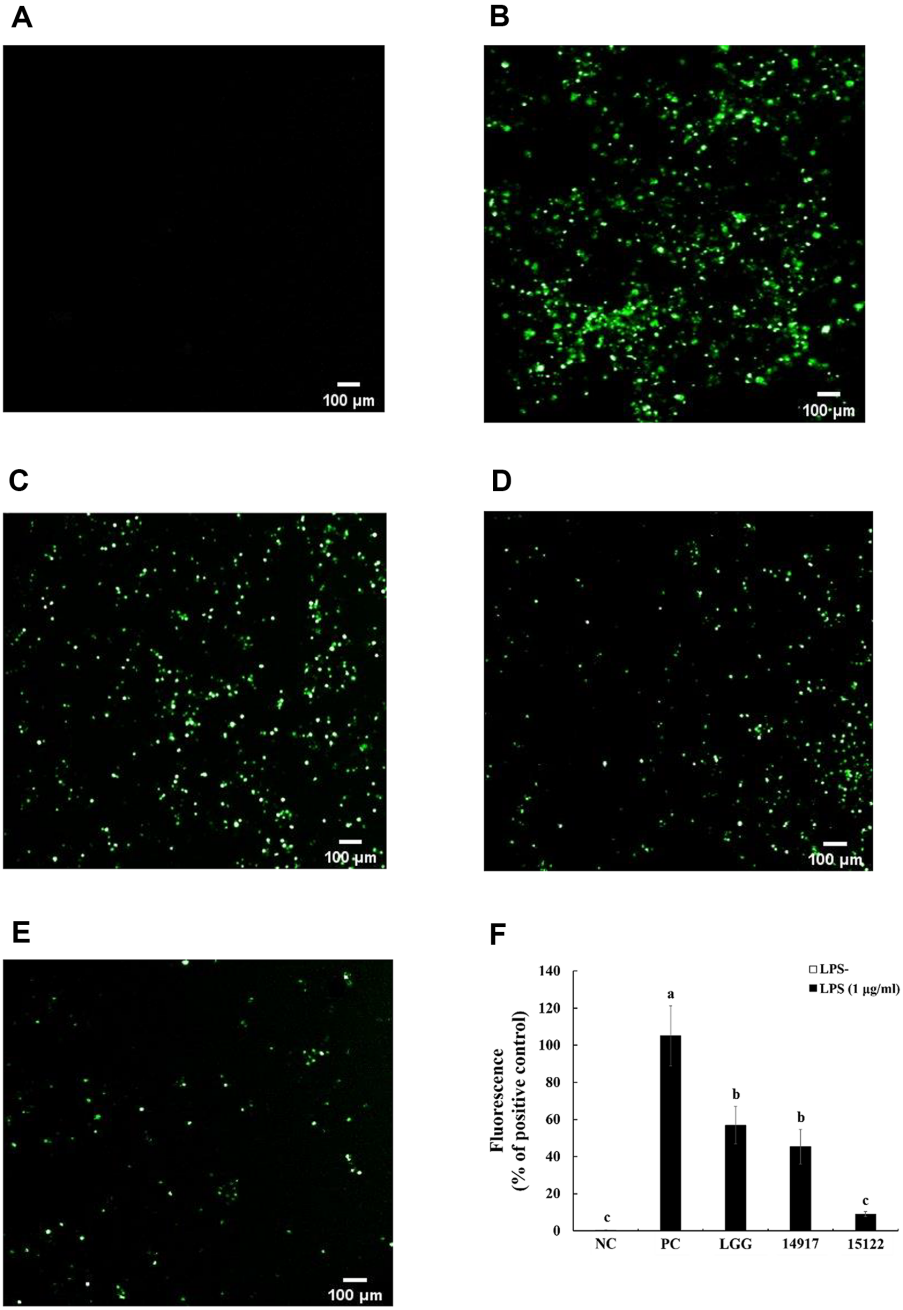
Effect of heat-killed LAB on ROS production in LPS-induced RAW 264.7 cells. (**A**) Negative control, (**B**) positive control, (**C**) LGG with LPS, (**D**) *L. plantarum* ATCC 14917 with LPS, (**E**) *L. plantarum* KU15122 with LPS, (**F**) quantification of ROS production. NC, negative control without LPS; PC, positive control with LPS; LGG, *L. rhamnosus* GG; 14917, *L. plantarum* ATCC 14917; 15122, *L. plantarum* KU15122. Data are presented as mean ± standard deviation of triplicate experiments. Different letters on error bars represent significant differences (*p* < 0.05).

The impact of heat-killed *L. plantarum* KU15122 on the generation of ROS in RAW 264.7 cells induced by LPS was assessed. ROS production increased dramatically upon stimulation with LPS (positive control; Fig. 6B). Pretreatment with heat-killed LAB markedly reduced ROS production; moreover, *L. plantarum* KU15122 displayed an alleviating effect similar to that in the LPS-negative group (Fig. 6A-6F).

### Bacterial EPS of *L. plantarum* KU15122 and Its Anti-Inflammatory Effect

**Fig. 7:**
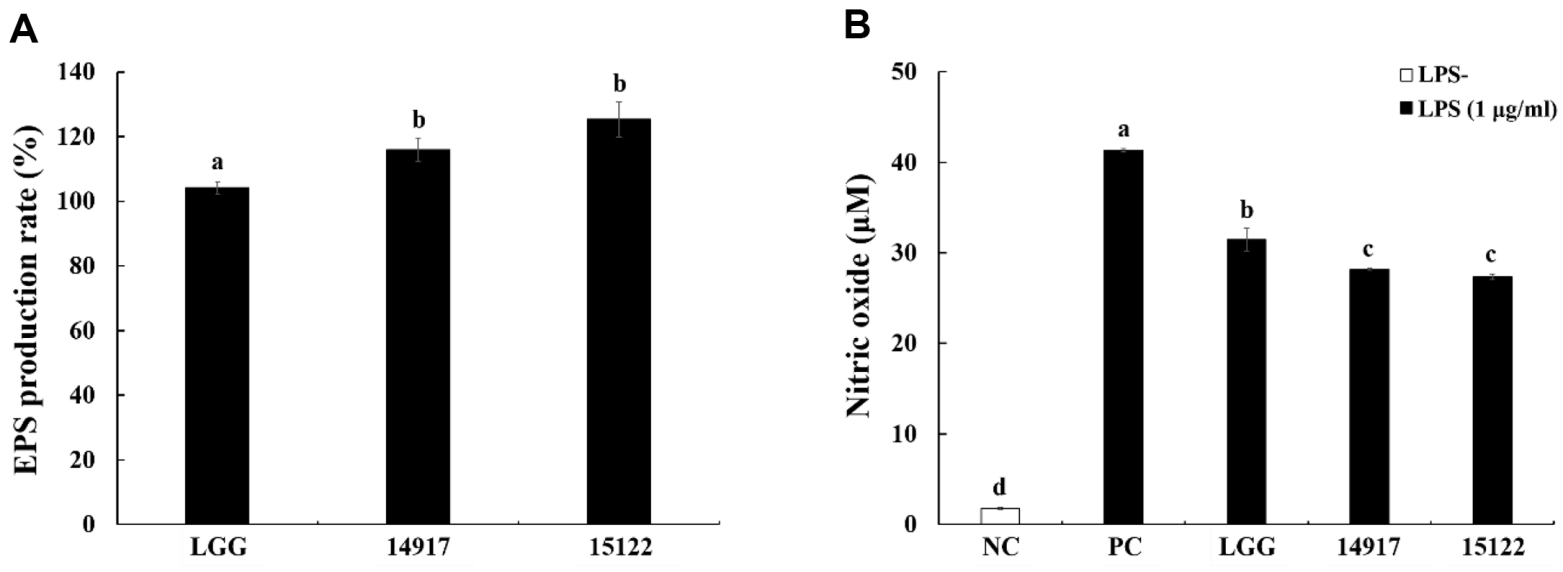
Total EPS production rate of LAB strains and its effect on NO production. (**A**) Total EPS production rate, (**B**) NO production. NC, negative control without LPS; PC, positive control with LPS; LGG, *L. rhamnosus* GG; 14917, *L. plantarum* ATCC 14917; 15122, *L. plantarum* KU15122. Data are presented as mean ± standard deviation of triplicate experiments. Different letters on error bars represent significant differences (*p* < 0.05).

*L. plantarum* KU15122 exhibited superior productivity compared with that of LGG and *L. plantarum* ATCC14917 (Fig. 7A).

MTT assays were conducted at different EPS concentrations (50, 100, 150, and 200 μg/ml), and no cytotoxicity was observed up to the maximum concentration of 200 μg/ml (data not shown). An NO assay using the extracted bacterial EPS showed results similar to those of paraprobiotics (Fig. 1). *L. plantarum* KU15122 had the best anti-inflammatory effect, considering that the amount of NO produced was lower than that produced by LGG and *L. plantarum* ATCC 14197. From these findings, it can be inferred that the anti-inflammatory impact of *L. plantarum* KU15122 might be due to EPS (Fig. 7B).

## Discussion

Extensive studies have been conducted on *L. rhamnosus* GG (LGG), a standard probiotic strain. *L. plantarum* ATCC 14917, recognized for its anti-inflammatory effects by suppressing inflammatory cytokines, has been suggested as a reference organism [2, 19]. Moreover, *L. plantarum* ATCC 14917 has shown beneficial effects in alleviating adipose inflammation [20] and preventing fatty liver disease [3]. The functionality of *L. plantarum* KU15122 was assessed by comparing it with strains known for their excellent functionality.

NO is a labile radical and a ROS consisting of one nitrogen atom covalently bonded to a single oxygen atom with an unpaired electron. Proinflammatory cytokines induce the production of iNOS in monocytes, macrophages, neutrophils, granulocytes, and various other cells during inflammatory reactions [21]. iNOS is induced in response to different agents, such as LPS or proinflammatory cytokines, through various signaling pathways [22]. Major cellular receptors, such as Toll-like receptors and CD14, regulate and modulate iNOS activity in macrophages [23]. Cell-free supernatant of *L. plantarum* WiKim0125 isolated from kimchi was decreased NO production and inflammatory cytokines, IL-1β, IL-6, and MCP-1 [24].

PGE_2_ serves various biological roles, including its active involvement in inflammation, where it facilitates local vasodilation, recruits, and activates inflammatory cells; it also act as an important marker of anti-inflammatory reactions, regulated by COX-2 [25]. Additionally, PGE_2_ has a significant impact on intestinal smooth muscle function in both healthy and diseased patients by causing contractions in small intestinal smooth muscle cells [26]. According to a previous research, the levels of PGE_2_ were found to correlate with the extent of inflammation and exhibited a repetitive pattern [26]. Therefore, it was anticipated that *L. plantarum* KU15122 possesses potential anti-inflammatory activity through inhibition of these proinflammatory cytokines (Figs. 2B and 3A).

The inflammatory cytokine TNF-α, alternatively referred to as cachectin, holds significance in certain pain models due to its pivotal role [28]. IL-1β is released during infection, inflammation, and cell injury by monocytes and macrophages and by nonimmune cells as well [29]. In addition, IL-6 signaling protein induces acute phase reactions in chronic diseases, typically those caused by immune stress [28]. According to previous *in vivo* studies, *L. plantarum* 299v has shown effectiveness in decreasing the histological assessments and levels of cytokines linked to IBD across different animal studies involving colitis [30]. Figs. 2 and 3 demonstrate that *L. plantarum* KU15122 effectively suppresses the generation of multiple inflammatory mediators and cytokines.

The primary regulatory transcription factor, NF-κB can form dimers, either by pairing with identical partners or with different ones such as p50 and p65 proteins. These dimers are initially held together by the inhibitor IkBα. The separation of these complexes is triggered by various factors, including cytokines, ultraviolet light, free radicals, stress, oxidized low-density lipoproteins, and bacterial and viral antigens [30]. AP-1 is another major TLR-mediated transcription factor. Phosphorylated MAPK, particularly JNK, can also activate c-Jun [33]. It was suggested that *L. plantarum* KU15122 reduces p-c-Jun and hampers the activation of the IKK-NF-κB signaling pathway, resulting in the formation of the AP-1 complex and a reduction in p65 nucleus entry in response to LPS stimulation. This lowers the production and release of inflammatory factors (Fig. 4A-4F).

Within the signaling network regulating cell growth and division, ERK, a member of the MAPK family, plays a crucial role. Inflammatory processes trigger the activation of the p38 and ERK signaling pathways, which have been shown to be critical in IL-6 production [33]. JNK has a role in the development and function of T cells, as well as the production of proinflammatory cytokines like IL-2, IL-6, and TNF-α [34]. Additionally, it was indicated that probiotics notably decreased the production of examined proinflammatory cytokines in cell culture, potentially by hindering the activation of the NF-κB and MAPK signaling pathways through TLR4 [35]. The anti-inflammatory activity of heat-killed *L. plantarum* KU15122 has been demonstrated by its ability to inhibit LPS receptors' expression of TLR-4-mediated MAPK signaling (Fig. 5A-5D).

Postbiotics like EPS, created by LAB, can engage with host cells as ligands, protecting the host by binding to pathogens in the gut [1]. Similarly, EPS of *L. plantarum* has been extensively studied regarding its biological functions, structure, and genes [36]. In addition to their use in pharmacology and nutraceuticals, EPS act as immunomodulatory, antimicrobial, antioxidant, cholesterol-lowering, anticancer, and prebiotic agents [37]. Kwon *et al*. [38] demonstrated that EPS has anti-inflammatory effects. As expected, *L. plantarum* KU15122 yielded the highest amount of extracted EPS compared with that of LGG and *L. plantarum* ATCC 14917. Further, using the extracted EPS for NO production experiments, *L. plantarum* KU15122 exhibited a markedly inhibition rate, indicating significant suppression. While comparing the anti-inflammatory effects of bacterial samples and EPS, which is known for its anti-inflammatory properties, similar experimental outcomes were observed. Therefore, it can be inferred that the anti-inflammatory effect of heat-killed bacteria is attributable to EPS (Fig. 7).

In conclusion, this present study demonstrates that *L. plantarum* KU15122, isolated from traditional Korean kimchi, exhibits notable anti-inflammatory property. Specifically, heat-killed *L. plantarum* KU15122 effectively reduced the production of NO and proinflammatory cytokines in RAW 264.7 cells when activated by LPS. Furthermore, in LPS induced murine macrophages, the impact of heat-killed *L. plantarum* KU15122 was evaluated through its effects on the expression of proinflammatory mediators and cell signaling pathways such as NF-κB, AP-1, and MAPK pathways. The experimental results clearly indicated a significant reduction in inflammation following treatment with heat-killed *L. plantarum* KU15122, suggesting its potential effectiveness in mitigating conditions characterized by inflammation such as IBD and IBS. The study findings suggest the potential use of heat-killed *L. plantarum* KU15122 as a preventive agent against inflammation.
